# Targeting Chronic Myeloid Leukemia Stem/Progenitor Cells Using Venetoclax-Loaded Immunoliposome

**DOI:** 10.3390/cancers13061311

**Published:** 2021-03-15

**Authors:** Mohammad Houshmand, Francesca Garello, Rachele Stefania, Valentina Gaidano, Alessandro Cignetti, Michela Spinelli, Carmen Fava, Mahin Nikougoftar Zarif, Sara Galimberti, Ester Pungolino, Mario Annunziata, Luigia Luciano, Giorgina Specchia, Monica Bocchia, Gianni Binotto, Massimiliano Bonifacio, Bruno Martino, Patrizia Pregno, Fabio Stagno, Alessandra Iurlo, Sabina Russo, Silvio Aime, Paola Circosta, Giuseppe Saglio

**Affiliations:** 1 Department of Clinical and Biological Sciences, University of Turin, 10043 Turin, Italy; Mohammad.houshmand@unito.it (M.H.); carmen.fava@unito.it (C.F.); 2 Molecular and Preclinical Imaging Centres, Department of Molecular Biotechnology and Health Sciences, University of Turin, 10126 Turin, Italy; francesca.garello@unito.it (F.G.); rachele.stefania@unito.it (R.S.); silvio.aime@unito.it (S.A.); 3 Division of Hematology, A.O. SS Antonio e Biagio e Cesare Arrigo, 15121 Alessandria, Italy; valentina.gaidano@unito.it; 4 Department of Hematology and Cell Therapy, A.O. Ordine Mauriziano, 10128 Turin, Italy; alessandro.cignetti@unito.it; 5 ADIENNE Pharma & Biotech SA, 6900 Lugano, Switzerland; michela.spinelli@adienne.com; 6 Center for Hematology and Regenerative Medicine, Karolinska Institutet, Department of Medicine, Karolinska University Hospital Huddinge, 14186 Stockholm, Sweden; mahin.nikougoftar.zarif@ki.se; 7 Department of Hematology, Clinical and Experimental Medicine, University of Pisa, 56126 Pisa, Italy; sara.galimberti@med.unipi.it; 8 Division of Hematology, ASST Grande Ospedale Metropolitano Niguarda, 20162 Milan, Italy; ester.pungolino@ospedaleniguarda.it; 9 Hematology Unit, Cardarelli Hospital, 80131 Naples, Italy; annun.mario@gmail.com; 10 Hematology Unit, Department of Clinical Medicine and Surgery, Federico II University, 80138 Naples, Italy; lulucian@unina.it; 11 School of Medicine, University of Bari ‘Aldo Moro’, 70121 Bari, Italy; giorgina.specchia@uniba.it; 12 Hematology Unit, Department of Medicine, Surgery and Neuroscience, Azienda Ospedaliera Universitaria Senese, University of Siena, 53100 Siena, Italy; monica.bocchia@unisi.it; 13 Unit of Hematology and Clinical Immunology, University of Padova, 35122 Padova, Italy; gianni.binotto@unipd.it; 14 Department of Medicine, Section of Hematology, University of Verona, 37134 Verona, Italy; massimiliano.bonifacio@univr.it; 15 Division of Hematology, Azienda Ospedaliera ‘Bianchi Melacrino Morelli’, 89124 Reggio Calabria, Italy; brunmartin@libero.it; 16 Hematology Division, Oncology and Hematology Department, AOU Città della Salute e della Scienza di Torino, 10126 Turin, Italy; ppregno@cittadellasalute.to.it; 17 Division of Hematology, Azienda Ospedaliero Universitaria Policlinico-Vittorio Emanuele Catania, 95124 Catania, Italy; fsematol@tiscali.it; 18 IRCCS Ca’ Granda—Maggiore Policlinico Hospital Foundation, 20122 Milan, Italy; alessandra.iurlo@policlinico.mi.it; 19 Division of Hematology, University of Messina, 98100 Messina, Italy; sabinarusso20@gmail.com

**Keywords:** chronic myeloid leukemia, leukemia stem cell, CD26, liposome, immunoliposome, targeted therapy, nanomedicine

## Abstract

**Simple Summary:**

Chronic myeloid leukemia stem cells (CML LSCs) are a rare and quiescent population that are resistant to tyrosine kinase inhibitors. CML LSCs have many features in common with hematopoietic stem cells (HSCs) and selectively targeting this population and sparing HSCs is of paramount importance. Targeted therapy by liposome via reducing side effects, controlled release, and versatile surface modifications is an effective way for the treatment of different cancers including leukemia. Here for the first time, we designed a liposome conjugated with Begelomab (anti-CD26) loaded with venetoclax to selectively target CD26+ CML LSCs/progenitor cells and to increase treatment outcome in CML patients. We proved that after antigen binding and drug release, the CD26+ LSCs/progenitor cells could be eliminated without any side effect on CD26− cells.

**Abstract:**

CML is a hematopoietic stem-cell disorder emanating from breakpoint cluster region/Abelson murine leukemia 1 (BCR/ABL) translocation. Introduction of different TKIs revolutionized treatment outcome in CML patients, but CML LSCs seem insensitive to TKIs and are detectable in newly diagnosed and resistant CML patients and in patients who discontinued therapy. It has been reported that CML LSCs aberrantly express some CD markers such as CD26 that can be used for the diagnosis and for targeting. In this study, we confirmed the presence of CD26+ CML LSCs in newly diagnosed and resistant CML patients. To selectively target CML LSCs/progenitor cells that express CD26 and to spare normal HSCs/progenitor cells, we designed a venetoclax-loaded immunoliposome (IL-VX). Our results showed that by using this system we could selectively target CD26+ cells while sparing CD26− cells. The efficiency of venetoclax in targeting CML LSCs has been reported and our system demonstrated a higher potency in cell death induction in comparison to free venetoclax. Meanwhile, treatment of patient samples with IL-VX significantly reduced CD26+ cells in both stem cells and progenitor cells population. In conclusion, this approach showed that selective elimination of CD26+ CML LSCs/progenitor cells can be obtained in vitro, which might allow in vivo reduction of side effects and attainment of treatment-free, long-lasting remission in CML patients.

## 1. Introduction

Chronic myeloid leukemia (CML) is a hematopoietic stem-cell disorder resulting from t(9;22)(q34;q11) that leads to juxtaposition of ABL1 on chromosome 9 and BCR on chromosome 22. The encoded protein triggers downstream signaling pathways by constitutive tyrosine kinase activity and causes enhanced proliferation of leukemic cells. The introduction of different generations of tyrosine kinase inhibitors (TKIs) revolutionized treatment strategy and increased treatment outcome in patients. In some CML patients who achieve deep molecular response (DMR) and maintain it for at least 2 years, therapy can be halted in a condition called treatment-free remission (TFR). In half of these patients, disease recurrence is detectable within 12 months of the treatment discontinuation, and therapy must be reinitiated. Several factors such as type and the duration of the treatment influence therapy outcome in CML patients [1].

One cause of TKIs resistance and relapse after therapy discontinuation is germane to the presence of a quiescent population called leukemia stem cells (LSCs). It has been displayed that CML LSCs are present at the time of diagnosis, during the treatment, and in patients who are in TFR. It seems these cells are not dependent on BCR-ABL1 tyrosine kinase activity and their stem-cell characteristics and BCR-ABL1 independent mechanisms guarantee their survival [2,3]. Due to their similarity to normal HSCs, CML LSCs lodge in the same microenvironment and make reciprocal interactions with cells in the bone marrow microenvironment (BMM). These interactions lead to the formation of the leukemic microenvironment and are accompanied by drug resistance, quiescence, and survival of leukemic cells [4]. Apart from protection by the BMM, activation of signaling pathways such as WNT/β catenin, Hedgehog, PI3K, JAK/STAT in a BCR-ABL dependent and independent mechanisms guarantee CML LSCs persistence and elimination of these cells solely by TKIs seems ineffective [2,5,6]. The presence of CML LSCs in patients under imatinib confirmed this phenomenon which can be counterweighed by higher dose or by more potent TKIs, at the cost of life-long complications [7]. A practical approach to target CML LSCs is to identify antigens that are selectively expressed. As CML LSCs reside in CD34+/CD38− fraction [8], a useful marker should, first, segregate normal from leukemic stem cells, and second, show lack or limited expression on more mature cells. Several markers have been proposed for the identification of CML LSCs including CD44, CD33, CD36, etc. but they are also expressed by normal stem/progenitor cells [9,10,11]. Other markers such as CD25, and IL-1RAP are significantly expressed by CML LSCs, but they might be detectable in normal progenitor cells [12,13,14,15]. It has been reported that CD26 (DPP4) is aberrantly expressed by CML LSCs/ progenitor cells and it has no expression on normal HSCs/progenitor cells population [16]. In a normal context, CD26 is expressed mainly by activated T cells and has a relation with the proliferation of these cells. In certain conditions including autoimmune diseases, lung adenocarcinoma, hepatocellular carcinoma, B-chronic lymphoblastic leukemia and, T acute lymphoblastic leukemia enhanced expression of CD26 is detectable. Previous reports claimed that inhibition of CD26 do not suppress activation and cytotoxic effect of T cells [17,18,19].

Currently, the emergence of nanomedicine-based therapy revolutionized treatment strategies in different cancers including leukemia. This type of treatment has many advantages over conventional forms of treatment such as reduction of side effects, controlled release, enhancement of blood half-life, and it might serve as an efficient carrier for combination therapy. Several factors define the eligibility of a nanocarrier for the in vivo application including stability, biocompatibility, biodegradability, and non-toxicity [20,21]. Most of the FDA-approved nanocarriers are liposome-based nanosystems that can incorporate many modifications and can be used for the delivery of both hydrophilic and hydrophobic agents. Using various compositions, the addition of polyethylene glycol (PEG) to increase blood circulation time and to avoid being phagocytosed by the immune system [22], and antibody conjugation for active targeting are part of these modifications [23].

Conventional forms of treatment and even approved nanomedicine therapies are based on the elimination of the bulk disease population and residual LSCs may circumvent the therapy [20,24]. Also, previous reports claimed that once LSCs face ineffective therapy they may evolve and change their characteristics [25]. So, the disappearance of LSCs in some CML patients during the treatment might be related to this phenomenon or the detection limit. Considering this, conjugating an antibody directed to an antigen that is solely expressed by LSCs to a carrier may increase the potency of the treatment and help us to target the root cause of the diseases.

It has been shown that BCL2 has a high degree of expression and activity in CML LSCs which further increases following the transformation of CML from chronic to blast phase. This higher activity is managed through both BCR/ABL dependent and independent mechanisms [26,27]. Previous reports proved that enhanced activity of BCL2 increases TKIs resistance [28]. Venetoclax is an anti-BCL2 inhibitor that targets the BH3 domain of BCL2, induces apoptosis in leukemic cells, and is widely used for the treatment of different hematological malignancies. As this agent might also target normal cells [29,30] selectively delivering to CML LSCs population is preferred. Meanwhile, venetoclax treatment reduced the frequency of long-term leukemia stem cells engraftment in BCR/ABL transgenic mice [31]. Although normal HSCs might be more dependent on MCL1 [32], normal progenitor cells, and more mature cells can be targeted upon venetoclax treatment. In different clinical trials administration of venetoclax led to neutropenia, thrombocytopenia, tumor lysis syndrome, and neutropenic fever [33,34]. Meanwhile, treatment of healthy bone marrow mononuclear cells with venetoclax reduced their cell viability and this toxic effect further increased by the addition of 5-azacitidine [35]. Furthermore, Ko et al. claimed the higher sensitivity of healthy CD34+ cord blood stem cells to venetoclax compared to CML cells [36]. Considering these, selectively delivering of venetoclax to the leukemic cell population may decrease toxicity on other normal cells.

Herein, to selectively target CML LSCs we designed a liposomal carrier conjugated with Begelomab (anti-CD26) loaded with venetoclax. By eradicating resistant cells, this strategy may help to increase the number of patients who achieve TFR, to minimize the drug side effects, and to maintain TFR for a longer period.

## 2. Materials and Methods

### 2.1. Patients Sample Collection

For this study, bone marrow (BM) and peripheral blood (PB) of newly diagnosed and TKI-resistant CML patients were collected as part of the Italian national study GIMEMA CML1415—Sustrenim, approved by the Ethical committee of the Coordinating Center on 11 May 2016. All samples were de-identified. Also BM samples of three healthy donors were collected after informed consent. Mononuclear cells (MNCs) were isolated from both BM and PB using Ficoll-Hypaque (Sigma Aldrich, Milan, Italy). MNCs were cultured in stemMACS media (Miltenyi Biotec, Bergisch Gladbach, Germany) supplemented with FLT-3L (100 ng/mL), SCF (100 ng/mL), IL-3 (20 ng/mL), and TPO (20 ng/mL). For all experiments that contain primary cells, fresh samples were used.

### 2.2. Flow Cytometry Analysis

To measure the percentage of CD26+ cells in newly diagnosed and resistant CML patients, a set of antibodies consisting of CD34-PE (Invitrogen, clone: QBEND/10), CD38−FITC (Invitrogen, Waltham, MA, USA, clone: HIT2), CD45-Super bright 436 (Invitrogen, Waltham, MA, USA, clone: 2D1), and CD26−APC (Miltenyi biotech, Bergisch Gladbach, Germany, clone: FR10-11G9) were used. In this experiment after the separation of MNCs, the abovementioned antibodies were added to 5 × 10^5^ cells and they were incubated for 30 min at 4 °C. Then, the percentage of CD26+ cells in CD45dim/CD34+/CD38+ and CD38− were measured by multi-color flow cytometry (FACSVERSE, (Becton, Dickinson and Company) BD-Bioscience, San Jose, CA, USA), and the results were analyzed using Kaluza software version 2.1 (Beckman Coulter Fullerton, Brea, CA, USA).

### 2.3. Cell Culture

CD26 positive cells including CMLT1 (CML cell line), MOLT4 (T-ALL cell line), cytokine induced killer cells (CIK), and negative cells for expression of CD26 consisting of HL60 (acute myeloid leukemia (AML) cell line), NB4 (AML cell line), K562 (CML cell line) were selected for the selectivity assay and to check the kinetic binding capacity. The expression of CD26 in these cell lines was analyzed before performing the experiments. CIK cells were generated from healthy donors as described previously [37]. All cells were cultured in RPMI1640 (Gibco, Thermo Fisher Scientific, Waltham, MA, USA) supplemented with 10% heat-inactivated fetal bovine serum (FBS) (Gibco, Thermo Fisher Scientific, Waltham, MA, USA) and 1% of penicillin/streptomycin (Gibco, Thermo Fisher Scientific, Waltham, MA, USA). Cell lines were kept in culture no longer than 5 weeks and regularly were tested for mycoplasma contamination.

### 2.4. Immunoliposome Preparation

Anti-CD26 immunoliposomes were formulated as follows: DPPC (1.2-Dipalmitoyl-sn-glycero-3-phosphocoline, Avanti Polar Lipids Inc., Alabaster, AL, USA)/Cholesterol/DSPE-PEG2000 (1.2 Distearoyl-sn-glycero-3-phosphoethanolamine-*N*-(methoxy(polyethy-leneglycol)-2000)Ammonium salt, Avanti Polar Lipids Inc., Alabama, USA)/DSPE-PEG2000-NHS (3-(*N*-succinimidyloxyglutaryl)aminopropyl, polyethyleneglycol-carbamyl distearoylphosphatidyl-ethanolamine, NOF Corporation, Tokyo, Japan) in ratio 74/20/5/1, 20 mg/mL. For confocal microscopy studies, Rhodamine-DOPE (1,2-dioleoyl-sn-glycero-3-phosphoethanolamine-*N*-(lissamine Rhodamine B sulfonyl) (ammonium salt) Avanti Polar Lipids Inc., Alabaster, AL, USA) was added in a 0.5 molar ratio (DPPC/Cholesterol/DSPE-PEG2000/DSPE-PEG2000-NHS/Rhodamine-DOPE molar ratio corresponding to 73.5/20/5/1/0.5). Liposomes were prepared via the film hydration method. Briefly, 20 mg of phospholipids were dissolved in chloroform and the solvent was evaporated under vacuum using a rotavapor. The film was then hydrated at 55 °C with 1 mL of HEPES Buffer (38 mM HEPES, 150 mM NaCl, pH 7.4), and the resulting suspension was sonicated twice (30 s, 50% power, corresponding to 35 W) in ice with an electronic sonopuls UW2070 sonicator tip (BANDELIN electronic GmbH & Co. KG, Berlin, Germany). The liposomes were then incubated overnight at room temperature with 3 mL of anti-CD26 antibody (Begelomab, 1.99 mg/mL, molar ratio antibody: outwardly exposed DSPE-PEG2000-NHS = 1:3) under gentle magnetic stirring. Begelomab is a murine IgG2B monoclonal antibody against CD26 property of ADIENNE SA (Lugano, Switzerland) manufactured by ADIENNE Srl (Caponago, Italy). Begelomab is a clinical grade product, which has been used in two phase I/II studies for the treatment of steroid refractory acute graft versus host disease (SR-aGvHD). The MCB, WCB, the unprocessed bulk, the purified bulk, the drug product, and the EOPC were characterized for absence of undesirable viruses, potentially infective, or pathogenic, for human EMA guideline n. CHMP/BWP/398498/2005 (Phase 1/2). Patients enrolled in the two prospective studies, were tested for the presence of human anti-mouse antibodies, at the end of treatment they were all found to be negative, suggesting low immunogenicty of Begelomab.

The day after newly formed immunoliposomes were purified from unbound antibody on a Superose 6 10/300 GL gel filtration column (GE Healthcare) equilibrated with HEPES buffer using a Fast Protein Liquid Chromatography (FPLC) system (an AKTA pure, Pharmacia, Sweden) n. Diluted immunoliposomes collected by FPLC were brought to the initial volume (1 mL) using vivaspin centrifugal concentrators (10,000 Da, centrifugation at 3500 rpm, 4 °C, until the desired volume was reached). Size and polydispersity index (PDI) were checked by dynamic light scattering (DLS) using a Malvern Zetasizer 3000HS (Malvern, UK) (sample dilution 1:100 in HEPES buffer). The concentration of the free antibody collected by FPLC was determined spectrophotometrically (6715 UV/Vis Spectrophotomether, Jenway, UK), by measuring the absorbance at 280 nm and plotting it into a calibration curve constructed with standard solutions of 0.01 to 0.4 mg/mL of antibody dissolved in HEPES buffer (correlation coefficient of R^2^ = 0.999). For performing different experiments, untreated cells of each cell type were used as control.

The binding efficiency of the antibody to the liposomes was then determined as follows:

(1) $$\%\;CD26\;binding=100-\;\frac{mg\;free\;antibody}{mg\;incubated\;antibody}$$

### 2.5. Selectivity Assay

To check the selectivity of our carrier CD26+ and CD26− cells were treated with Rhodamine-conjugated immunoliposomes (IL-Rho). 1 × 10^4^ cells were plated in 96 wells plate and after the addition of immunoliposome (40 µL/mL), the selectivity was measured by flow cytometry analysis after 4 h of treatment. Meanwhile, cells were labeled with Carboxyfluorescein succinimidyl ester (CFSE) (Molecular Probes), were treated with IL-Rho and were analyzed by confocal microscopy. To check the selectivity of this system on primary cells, patient samples were sorted with FACSAria Ⅲ cell sorter (BD Biosciences, San Jose, CA, USA) based on CD45dim/CD34+/CD38−/CD26− for HSCs and CD45dim/CD34+/CD38−/CD26+ for CML LSCs. After the sorting, cells were cultured in StemMACS media (Miltenyi Biotec, Bergisch Gladbach, Germany) supplemented with the abovementioned growth factors and were treated with the designed IL-Rho for 4 h. The selectivity assay was carried out using flow cytometry and confocal analysis.

### 2.6. Kinetic Binding Assay

The kinetic binding assay was carried out using CD26+ (CIK) and CD26− (K562) cells. To this aim, 40 µL/mL IL-Rho were added to 1 × 10^4^ cells that were cultivated in 96 wells plate. Subsequently, the percentage of positive cells for Rhodamine after 1 h, 2 h, and 4 h of the treatment were measured by flow cytometry analysis.

### 2.7. Drug Loading and Stability

The antineoplastic agent venetoclax was dissolved in chloroform/methanol (1:1) at a concentration of 0.25 mg/mL. To obtain venetoclax-loaded immunoliposomes (IL-VX), 0.09 mg venetoclax (360 µL) were added to the phospholipid blend at the first step of liposome preparation, and then the solvent was evaporated under vacuum using a rotavapor and liposome preparation proceeded as reported above. After overnight incubation with the anti-CD26 antibody at room temperature (RT), immunoliposomes were separated from the free antibody and non-internalized venetoclax by AKTA purifier as described above.

To determine the encapsulation efficiency of venetoclax in immunoliposomes, 100 µL aliquot of immunoliposome was dried and then mixed with 200 µL of acetonitrile. The sample was sonicated for 10 min, centrifuged at 10,000 rpm for 10 min and the supernatant was analyzed by Waters Alliance 2695 HPLC system with Waters 2998 Photodiode Array (PDA) Detector, using a SunFire C18 Column, 100 Å, 3.5 µm, 4.6 mm × 150 mm and 0.1% TFA in water (solvent A) and 0.1% TFA in acetonitrile (solvent B). Elution was done with a linear gradient of (% mobile phase B/min) 25/0.1, 75/8.0, 98/8.1, 98/15, 100/1.0. at a 1 mL/min flow rate. The detection wavelength was set at 254 nm. Sample solution was injected at a volume of 25 μL. The RP-HPLC was calibrated with standard solutions of 0.5 to 50 μM (0.4 to 40 µg/mL) of venetoclax dissolved in acetonitrile (correlation coefficient of R^2^ = 0.9999). The encapsulation efficiency was defined by the ratio of measured and initial amount of venetoclax added during liposome preparation. To perform stability tests, 2 mL of IL-VX were placed in a dialysis bag (MWCO, 10 kDa). The samples underwent dialysis against 1 L of HEPES buffer at 37 °C with slight agitation via mechanical stirring. At various time intervals (0, 4, 24, 48, 72 h), 100 µL of sample was collected for residual venetoclax quantification by RP-HPLC as described above.

### 2.8. Apoptotic Assay

CMLT1 as CD26+ cells and HL60 as CD26− cells were treated with free venetoclax and IL-VX. 1 × 10^4^ cells were plated in 96 wells plate and treated with different concentrations of the free venetoclax (ranging from 10 nM to 1 µM) and IL-VX (ranging from 10 nM to 1 µM). After 48 h, an apoptosis assay by Annexin-V kit (Miltenyi Biotech, Bergisch Gladbach, Germany) was performed according to manufacturer protocol. Briefly, cells were collected and washed with Annexin buffer and after addition of Annexin-V FITC were incubated at RT for 15 min in the dark. Afterward, cells were washed again with Annexin buffer and Propidium Iodide (PI) was added. Apoptosis assays were acquired by FACSVERSE (BD-Bioscience) and were analyzed by Kaluza software version 2.1 (Beckman Coulter Fullerton, Brea, CA, USA).

### 2.9. Mitochondria Membrane Potential Assay

CMLT1 cells were treated for 48 h with 100 nM of IL-VX. Then, cells were collected and washed with cell culture medium. Next, cells were resuspended in cell culture medium, and 1 µL of tetramethylrhodamine methyl ester (TMRM) dye was added and they were incubated at 37 °C for 30 min. Following the washing step, cells were resuspended in 500 µL of cell culture medium and were analyzed by flow cytometry.

### 2.10. Cell Growth Assay

The growth rate of CD26+ and CD26− cells, were evaluated by cell counting kit-8 (CCK8) (Microtech, Naples, Italy). This kit works based on the reduction of dehydrogenase and the formation of formazan. The amount of produced formazan represents the number of live cells. First, 1 × 10^4^ CMLT1 and HL60 were added to 96 wells plate and various concentrations of IL-VX (ranging from 10 nM to 1 µM) were added. After 48 h, 10 microliters/well of CCK8 dye were added and cells were incubated for 3 h at 37 °C. After this step, absorbance at 450 nm was measured using GlowMax (Promega, Madison, WI, USA) instrument. In this experiment, untreated CMLT1 and HL60 were used as the control to obtain the percentage of cell growth following IL-VX treatment. Meanwhile, culture media was used as the background. To obtain the percentage of cell growth following protocol was used.

(2) $$\%\;cell\;growth=\frac{OD\;Treated\;cells-OD\;background}{OD\;untreated\;cells-OD\;background}\;\times 100$$

### 2.11. Cell Cycle Analysis

The cell cycle was measured using Vybrant DyeCycle orange stain kit (Thermo Fisher). After the treatment of 1 × 10^4^ CMLT1 and HL60 cells with different concentrations of IL-VX (ranging from 10 nM to 500 nM) for 48 h, cells were collected, washed with Phosphate buffered saline (PBS), and 0.4 µL of the dye were added per each condition. Then, cells were incubated at 37 °C for 30 min, were acquired by FACSVERSE and data were analyzed with Kaluza software.

### 2.12. Combination Treatment

We treated CMLT1 cells with different concentrations of IL-VX, imatinib (IM), nilotinib (NIL), and half-maximal inhibitory concentration (IC50) for each compound were obtained using Graphpad Prism software. Next, cells were treated for 48 h with each compound and the combination of IL-VX+IM and IL-VX+NIL based on their IC50. Finally, a combination index (CI) was obtained using CalcuSyn software.

### 2.13. Targeting CML LSCs Primary Cells

MNCs of newly diagnosed CML samples, (1 × 10^4^ cells) were plated in 96 wells plate and were cultured in StemMACS media supplemented with growth factors. Cells were treated by IL-VX with a venetoclax concentration of 100 nM. Then, cells were collected and washed with PBS and were labeled with antibodies against CD45, CD34, CD38, and CD26. Due to the sensitivity of primary cells to VX in comparison to cell lines, the experiment was carried out after 24 h of IL-VX treatment. The percentage of CD26 in treated and untreated cells in the stem/progenitor cells population was measured using flow cytometry analysis.

### 2.14. Statistical Analysis

Statistical analysis was carried out using GraphPrism software, version 8.0 (GraphPad Software, San 590 Diego, CA, USA). All data are reported as means ± SD with three independent experiments. Two-tailed paired Student’s t-test was used to assess differences between the two groups and *p* < 0.05 was considered significant. (* *p* < 0.05, and ** *p* < 0.01).

## 3. Results

### 3.1. CD26+ Cells Are Present in Newly Diagnosed and Resistant CML Patients

**Figure 1:**
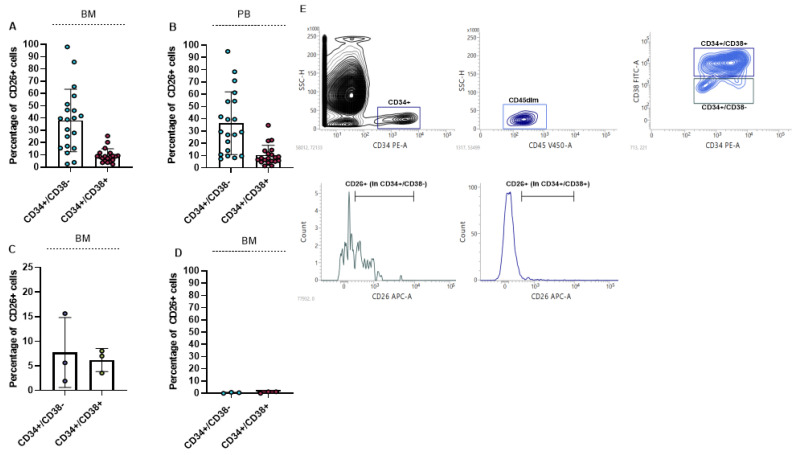
Percentage of CD26+ cells in newly diagnosed and resistant CML patients. (**A**,**B**) show percentage of CD26+ cells in CD45dim/CD34+/CD38− (stem cells) and CD45dim/CD34+/CD38+ (progenitor cells) in BM and PB of newly diagnosed CML patients, respectively. CD26+ cells are also detectable in CML patients to resistant TKIs in both stem cells and progenitor cells compartment (**C**). CD26 was not detectable in stem/progenitor cells compartment of healthy donors (**D**). Flow cytometry graph and gating strategy of one newly diagnosed patient is depicted in (**E**).

We measured the percentage of CD26 in BM and PB of 20 newly diagnosed CML patients. For the measurement of CD26 expression 5 × 10^5^ cells were acquired. Our results showed that CD26 is significantly expressed in CD45dim/CD34+/CD38− cells as stem cells compartment, and to a lesser extent in CD45dim/CD34+/CD38+ cells as progenitor cells population. The mean percentage of CD26+ in the stem-cell compartment of BM and PB was 38.07% (ranging from 2.61% to 98.02%) and 36.50% (ranging from 7.41% to 94.94%), respectively. Also, the mean percentage of CD26+ in the progenitor cell population in BM and in PB was 9.3% and 10.3% respectively. The percentage of CD26+ cells in BM and PB of newly diagnosed patients is displayed in Figure 1A,B. We also measured the expression of CD26 in BM of 3 TKIs resistant patients and we found that TKIs administration can significantly reduce LSCs population but is not able to eliminate all the CD26+ cells. In all 3 resistant patients CD26+ cells were detectable in both stem cells and progenitor cells population (Figure 1C). Meanwhile, stem cells/progenitor cells compartment of three healthy donors did not express CD26 and this result confirmed previous findings [16,38] (Figure 1D). The flow cytometry graph of one newly diagnosed patient is shown in Figure 1E. This graph also represents the gating strategy that was used to measure the expression of CD26 in patients.

### 3.2. Characterization of the Immunoliposome

The average diameter of immunoliposome was measured by DLS and corresponded to 145 ± 20 nm with a PDI of 0.15 ± 0.06. Antibody (Begelomab, ADIENNE, Lugano, Switzerland) was conjugated on the lipo-some surface through an amide linkage occurring between the NHS group of PEG (DSPE-PEG2000-NHS) and the ɛ-amine of lysine residues of antibodies. The amount of bounded antibody was quantified, indirectly, by measuring the absorbance at 280 nm of free antibody collected during size exclusion chromatography of liposome-antibody reaction mixture. The results indicated a concentration of antibody of 1 mg/mL in the liposome suspension corresponding to 16.6 ± 0.2% of conjugation efficiency.

### 3.3. ILs Selectively Targets CD26+ Cells

**Figure 2:**
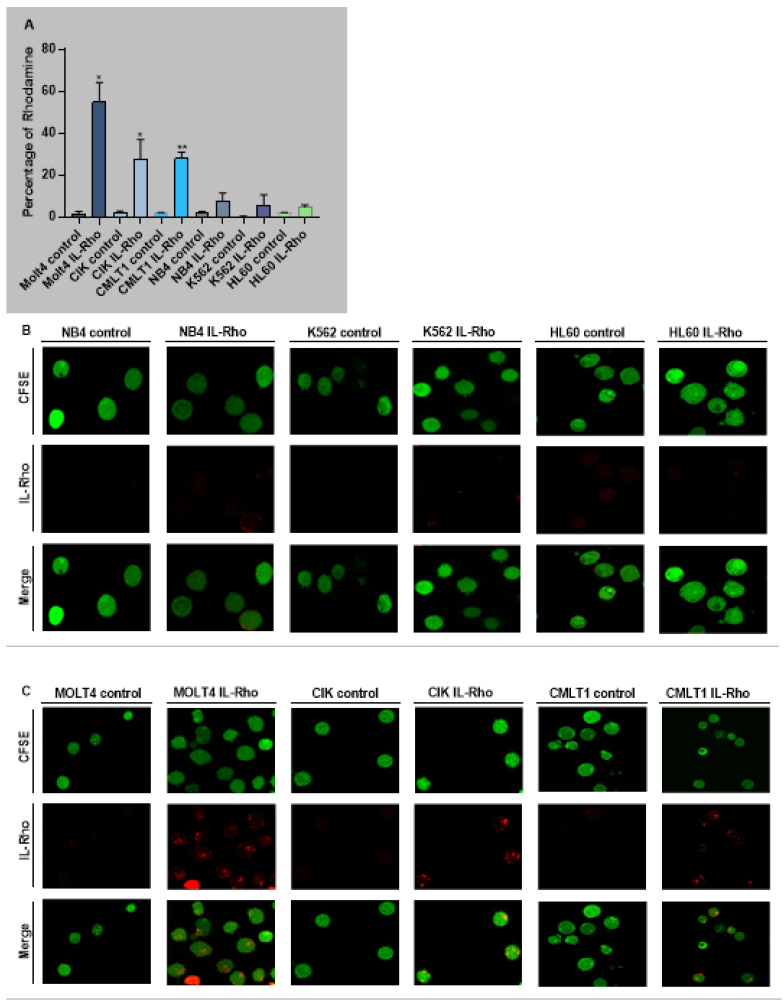

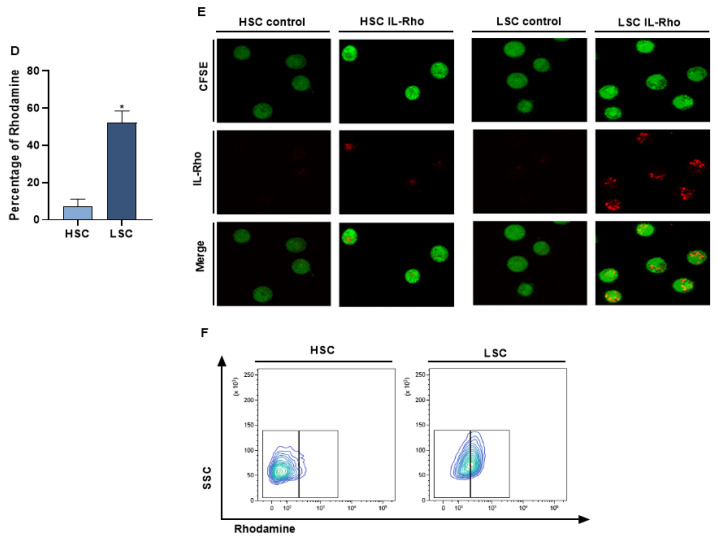
Selectivity assay in CD26+ and CD26− cells. (**A**) represent the percentage of Rhodamine in CD26+ and CD26− cells that were acquired after 4 h of the treatment by IL-Rho. Each treated cell line was compared with untreated cells as control. As is clear in (**B**) IL-Rho is not detectable in CD26− cells while the presence of IL-Rho in CD26+ cells (**C**) is visible (63× magnification). Also, percentage of Rhodamine was measured by flow cytometry in sorted newly diagnosed CML sample based on CD45dim/CD34+/CD38−/CD26− (HSCs) andCD45dim/CD34+/CD38−/CD26+ (LSCs) expression (**D**). As is displayed in (**D**) IL-Rho remarkably targeted CML LSCs, and this result was confirmed by confocal analysis as shown in (**E**) (63× magnification). Flow cytometry graph of one sorted newly diagnosed CML patient treated with IL-Rho is depicted in (**F**). (* *p* < 0.05, and ** *p* < 0.01).

The selectivity of our system in targeting CD26+ cells (CIK, MOLT4, CMLT1) and sparing CD26− cells (K562, HL60, NB4) was measured following treatment with IL-Rho. Our results showed that after 4 h of the treatment, IL-Rho selectively targeted CD26+ cells and spared CD26 negative cells (Figure 2A). To confirm the results of flow cytometry analysis, following labeling cells with CFSE and 4 h of treatment with IL-Rho, the selectivity was investigated by confocal microscopy. As expected, and is displayed in Figure 2B, there was no or little Rhodamine signal in K562, HL60, and NB4 cells as they are CD26 negative cells, while IL-Rho was clearly detectable in CD26+ cells (Figure 2C). After this step, primary CML cells from newly diagnosed patients were sorted based on CD45dim/CD34+/CD38−/CD26− expression (HSCs) and CD45dim/CD34+/CD38−/CD26+ expression (CML LSCs) and were treated with IL-Rho. After 4 h of treatment with IL-Rho, our result confirmed that the designed system could selectively target CML LSCs while HSCs showed minor reaction with IL-Rho (Figure 2D). Confocal microscopy image analysis revealed that the developed IL-Rho selectively interacted with CML LSCs while sparing HSCs (Figure 2E). Flow cytometry graph of a sorted CML cells treated with IL-Rho is depicted in Figure 2F.

### 3.4. Kinetic Binding Assay of Immunoliposome

**Figure 3:**
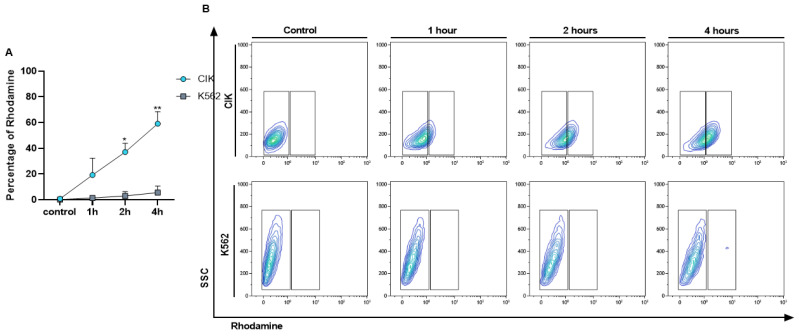
Kinetic binding assay. Interaction of IL-Rho after 1 h, 2 h, 4 h of the treatment with CD26+ CIK cells and CD26− K562 cells was measured by flow cytometry. The Rhodamine percentage in positive and negative cells for CD26 is displayed in (**A**) and flow cytometry graph of the kinetic binding assay is shown in (**B**). (* *p* < 0.05, and ** *p* < 0.01).

To check the interaction of immunoliposome with CD26 positive and negative cells along time, a kinetic binding assay was performed. After confirming the selectivity of IL-Rho, we also demonstrated that the percentage of interaction increased in a time dependent manner. The median percentage of Rhodamine positive cells was 19.19% at 1 h, 37.03% at 2 h, and 59.07% at 4 h in CIK cells but remained negative in K562 cells (Figure 3A,B). This result also proved that a prolonged time of immunoliposome treatment does not induce unspecific binding to CD26 negative cells.

### 3.5. IL-VX Significantly Induces Apoptosis in CD26+ Cells

**Figure 4:**
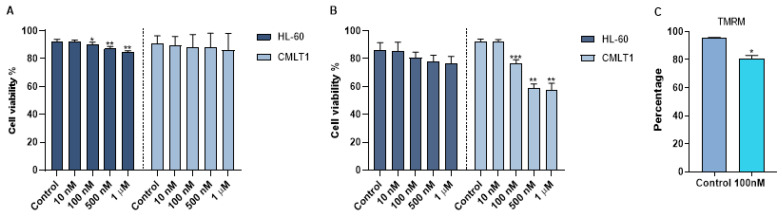
Apoptosis assay following IL-VX and free venetoclax treatment. In (**A**) treatment of CD26+ CMLT1 cells with different concentrations of free venetoclax did not induce any apoptotic effect and just a minor apoptotic effect was seen in HL60 as CD26− cells. However, IL-VX significantly enhanced apoptosis in CMLT1 starting from 100 nM while sparing CD26− HL60 cells (**B**). Reduction of TMRM fluorescent signal (which indicates reduction of mitochondria membrane potential) was seen in CMLT1 treated with 100 nM of IL-VX (**C**). This result confirms the apoptotic effect of IL-VX in CD26+ CMLT1 cells. (* *p* < 0.05, ** *p* < 0.01 and *** *p* < 0.001).

To obtain a therapeutic effect, venetoclax was loaded into immunoliposomes. The size of IL-VX was the same of IL-Rho (around 145 nm). To quantify the efficiency of encapsulation, an RP-HPLC method was established with good linearity ranges of 0.4 to 40 µg/mL (y = 29,699x + 4044.4; R^2^ = 0.9999) at 254 nm. The retention time of venetoclax was 8.03 min (Figure S1). The venetoclax encapsulation efficiency was 6.4 ± 0.1% corresponding to a concentration of 6.0 µg/mL of venetoclax. The in vitro stability of IL-VX was investigated in HEPES buffer at 37 °C by RP-HPLC measurements of samples placed in a dialysis bag. The cumulative percentage release is shown in Figure S2. Data obtained displayed high stability of venetoclax entrapment in the liposomes (~6% VX released after 72 h at 37 °C), as already reported for other drugs encapsulated in nanosystems with similar composition. After loading immunoliposome with venetoclax, we used different concentrations of IL-VX including 10 nM, 100 nM, 500 nM, and 1 µM for the treatment of CMLT1 and HL60 cells. To study the selectivity of IL-VX and to demonstrate the higher efficiency of the venetoclax-loaded liposome, the cells were also treated with free venetoclax at the same doses. Our results displayed that after 2 days of the treatment, free venetoclax did not have any apoptotic effect on CMLT1 cells even at the concentration of 1 µM, and a higher concentration was needed to observe the cytotoxic effect in this cell line (Figure 4A). However, it slightly reduced cell viability in HL60 cells. On the contrary, IL-VX induced its toxic effect on CD26+ CMLT1 cells starting from 100 nM, while its effect on CD26− HL60 cells was not significant (Figure 4B). These results confirmed the selectivity and efficiency of IL-VX and offered the opportunity to eliminate resistant cells even with a lower dose of the drug. As is clear in Figure 4C, TMRM percentage in CMLT1 was reduced following treatment with IL-VX. In healthy cells, TMRM accumulates in mitochondria and displays a bright signal. Reduction of TMRM signal may represent the reduction of mitochondria membrane potential and the start of apoptosis in treated cells.

### 3.6. IL-VX Reduces Cell Growth in CD26+ Cells

**Figure 5:**
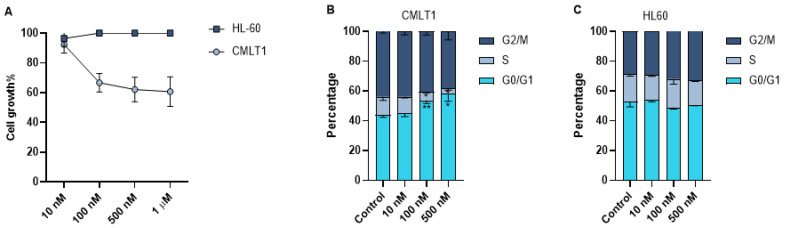
Cell growth assay and cell cycle analysis. Treatment with different concentrations of IL-VX starting from 100 nM to 1 µM could significantly decrease cell growth in CD26+ CMLT1 cells while it did not affect cell growth of CD26− HL60 cells (**A**). Meanwhile, cell cycle analysis confirmed the cell growth experiment, and an arrest in G0/G1 was seen in CMLT1 (**B**). Cell cycle remained unchanged in CD26− HL60 cells (**C**). (* *p* < 0.05, and ** *p* < 0.01).

As leukemic cells are highly proliferative, we also measured the effect of IL-VX on cell growth of CMLT1 and HL60. Our results showed that treatment with different concentrations of IL-VX has a negative effect only on the cell growth of CD26+ CMLT1 cells. IL-VX even at high concentrations, could not inhibit HL60 growth rate (Figure 5A).Although our results showed apoptosis induction as the main mechanism of action of IL-VX, we also measured the cell cycle of live cells. Our results showed that treatment of CMLT1 with various concentrations of IL-VX starting from 100 nM resulted in G0/G1 arrest (Figure 5B) in live cells and inhibited their proliferation. Cell cycle in CD26− HL60 cells remained intact, confirming the selectivity of IL-VX (Figure 5C).

### 3.7. IL-VX Demonstrates Synergy with TKIs

**Figure 6:**
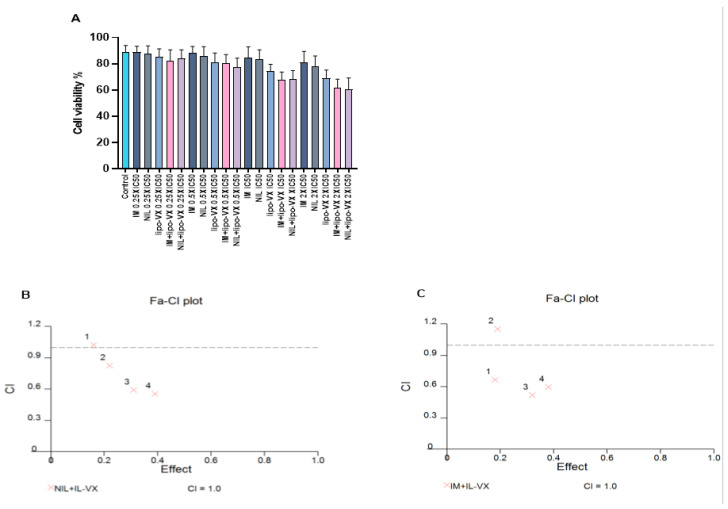
Combination treatment of IL-VX with imatinib and nilotinib. Treatment of CMLT1 with IL-VX, NIL, IM, and a combination of IL-VX with NIL and IL-VX with IM was performed based on their IC50 (**A**). As is displayed in (**B**) the combination of 0.50 × IC50, IC50, and 2 × IC50 of IL-VX and NIL had a synergistic effect and we had also synergistic effect between IL-VX and IM in 0.25 × IC50, IC50, 2 × IC50 (**C**). Synergistic effect (CI < 1), additive effect (CI = 1), antagonistic effect (CI > 1).

To further enhance the effectiveness of the IL-VX therapy and to target most of the leukemic cell population, we performed a combined treatment with TKIs. After treatment of CMLT1 with different concentrations of IL-VX, NIL, and IM we obtained the IC50 of ILVX (12.54 nM), NIL (5.4 nM), and IM (68.68 nM). After this step, we used 0.25 × IC50, 0.5 × IC50, IC50, and 2 × IC50 of the abovementioned compounds for combined treatment (Figure 6A). Although each compound was able to reduce cell viability in CMLT1 mostly at higher doses, our results showed that IL-VX and NIL at 0.5 × IC50, IC50, and 2 × IC50 (Figure 6B) and with IM at 0.25 × IC50, IC50, and 2 × IC50 (Figure 6C) have higher toxicity with CI < 1. These results indicate a strong synergism between IL-VX and TKIs for the treatment of CML and might give the opportunity to further reduce in vivo dose of drugs to induce apoptosis in leukemic cells.

### 3.8. IL-VX Remarkably Reduces CD26+ CML LSCs/Progenitor Cells in Patient Samples

**Figure 7:**
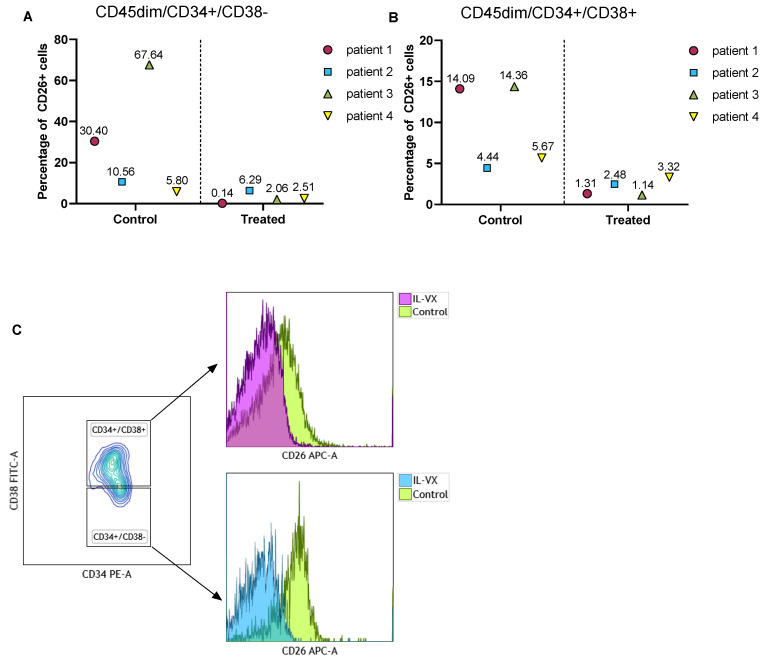
Treatment of primary CML samples with IL-VX. Following treatment of MNCs from four newly diagnosed CML samples with 100 nM IL-VX, a significant reduction of CD26+ cells in CD45dim/CD34+/CD38− stem cells population and in CD45dim/CD34+/CD38+ progenitor cells was recorded (**A**,**B**). Flow cytometry graph of one newly diagnosed patient sample treated with 100 nM IL-VX is displayed in (**C**), where reduction of CD26+ cells in both stem cells and progenitor cells fraction is evident.

To verify the toxicity of IL-VX on CML LSCs, we treated MNCs of 4 different newly diagnosed CML patients with IL-VX. Following this step, we measured the percentage of CD26 in the stem/progenitor cells population. Although the percentage of CD26 was variable at baseline, IL-VX at a concentration of 100 nM could eliminate CD26+ cells in all cases (Figure 7A,B). Selectively targeting CD26+ cells in CML patients is a viable option to avoid targeting normal HSC/progenitor cells. Flow cytometry data of one patient treated with IL-VX is depicted in Figure 7C.

## 4. Discussion

For decades, treatment strategy in leukemia has relied on the administration of chemotherapy agents that target the bulk disease population, resulting in numerous side effects. In recent years, our extensive knowledge about leukemia development shifted the treatment plan toward targeted therapy [39]. In this study, by developing a new form of targeted therapy we aimed to eliminate CD26+ CML LSCs/progenitor cells selectively and to reduce side effects on CD26− cells.

CD26 has a cleavage activity against diverse substrates such as chemokines, such as disruption of the SDF1-CXCR4 axis leading to the release of CML LSCs into the PB [16]. It has been proved that the percentage of this antigen has a direct correlation with white blood cell count, but its relationship with risk-stratification in patients was not confirmed [3,16,40]. Based on previous reports this antigen is detectable in all newly diagnosed CML patients, but the expression rate varied from patient to patient [3,16,40]. In line with these notions, we showed that CD26+ cells can be measured in all newly diagnosed patients. Although CD26 was significantly expressed by CD34+/CD38− cells, it was also detectable at a lower degree in the CD34+/CD38+ fraction. Meanwhile, in our experiments, we could not find any difference between PB and BM for CD26 expression, suggesting that PB samples can be used for measurement of residual leukemic cells. CD26 is also detectable in CML patients who are under TKIs treatment and in patients who are in TFR [3]. It has been claimed that CD26+ CML LSCs are the most insensitive population to TKIs [41]. Here, we also confirmed that the mean percentage of CD26+ CML LSCs in resistant patients was lower compared to newly diagnosed patients but CD26+ cells were still detectable in both stem/progenitor cells population. Therefore, selectively targeting these residual leukemic cells and sparing normal HSCs/progenitor cells is of paramount importance. Apart from CML LSCs, the same strategy might be applicable for targeting LSCs in AML patients with FLT3-ITD mutation and Philadelphia positive acute lymphoblastic leukemia that express CD26 [38,42].

Liposome treatment of leukemia with Vyxeos and Marquibo does not benefit from active targeting, thus not being able to segregate normal and leukemic cells. Moreover, previous studies that employed nanoparticle conjugated antibodies to target LSCs demonstrated promising results [43,44,45]. In this study, we conjugated a monoclonal antibody called Begelomab that has been used for the treatment of acute graft versus host disease (aGvHD) [46] to a PEGylated liposome. After antibody conjugation, we checked the selectivity of our system in cell lines and CML primary cells and our results confirmed that we could selectively target CD26+ cells. Our results showed that IL-Rho did not target normal HSCs, but it could target CML LSCs and CIK cells. Although CD26 is not expressed by normal HSCs/progenitor cells, an up-regulated expression by some mature cells such as activated T cells is detectable [47]. It has been reported that CD26 CAR-T cells showed a fratricide killing and an off-tumor cytotoxicity against activated T cells due to the CD26 expression. Although CD26 CAR-T cells exhibited cytotoxic activity in LSCs, they showed a delay in in vitro expansion and white blood cells reduction in immunocompetent mice. At the contrary IL does not exhibit fratricide killing but it may cause partial T cell depletion. However, the activity and function of immune cells can be restored through various strategies [48,49]. The reason CML LSCs/progenitor cells express CD26 has not been defined yet, but it might be related to the BMM modifications [4].

As Begelomab alone had no toxicity in treated cells, loading the carrier with an appropriate agent to eliminate leukemic cells was essential. Based on previous reports that confirmed the efficiency of BCL2 inhibition in eliminating CML LSCs, we loaded this carrier with venetoclax. In our experiment, IL-VX not only eliminated CD26+ cells but also reduced the drug concentration that was required to induce apoptosis in leukemic cell lines. This strategy, via recognizing specific antigens on the surface of the targeted cells and reduction of off-target effects, demonstrates higher efficiency in comparison to free drug and passive targeting. Venetoclax is a substrate of the p-glycoprotein pump and liposome delivery allows for bypassing this pump through endocytosis and may results in improved drug uptake and drug concentration within tumor cells [30,50]. In our study, free venetoclax could not induce apoptosis in our cells even at high concentrations while IL-VX introduced selectivity and high apoptotic rate in leukemic cells. This apoptotic effect was accompanied by cell growth arrest in CD26+ cells as a consequence of venetoclax treatment [51,52]. It has been shown that the addition of venetoclax to TKIs effectively targeted CML LSCs and reduced cell engraftment in mice [31]. We also proved that venetoclax and TKIs have a synergistic effect in CML cells and this combination strategy is a practical option for targeting both BCR/ABL dependent and independent mechanisms. The use of venetoclax in combination with TKIs for treatment of CML patients in chronic and blastic phase is being explored in clinical trials (NCT02689440, NCT03576547, NCT04188405). Meanwhile, treatment with TKIs plus IL-VX might be able to effectively target bulk disease population and residual disease burden at the same time, with reduced drug concentrations and lower side effects. Also, reduction of CD26+ cells in patient samples after 24 h of IL-VX treatment showed that this system works efficiently. Despite variable degree of CD26 expression, IL-VX could significantly target CD26+ cells in fresh samples from patients. The main advantage of this form of therapy is its precision to hit the target. So, we proved that after drug release, CML LSCs will be eliminated without any side effects on normal HSCs/progenitor cells. Also, this system reduces the amount of the drug that is needed to eliminate leukemic cells. This strategy may help us to increase the number of patients attaining and maintaining TFR without relapsing.

## Data Availability

Experimental data are available from the authors upon reasonable request and with permission of the University of Turin.

## Supplementary Materials

The following are available online at https://www.mdpi.com/2072-6694/13/6/1311/s1, Figure S1: HPLC chromatogram of venetoclax supernatant extracted from immunoliposome at 254 nm, Figure S2: % Venetoclax cumulative release from IL-VX measured at 37 °C, at different time points.
