# Supplementary material for: Targeting Chronic Myeloid Leukemia Stem/Progenitor Cells Using Venetoclax-Loaded Immunoliposome
Source: Cancers (Basel). 2021 Mar 15;13(6):1311. doi: 10.3390/cancers13061311 (PMC8000981; doi:10.3390/cancers13061311)
Supplement: Supplementary file 1 [file cancers-13-01311-s001.pdf]

Targeting chronic myeloid leukemia stem/progenitor cells using venetoclax loaded immunoliposome

Mohammad Houshmand, Francesca Garello, Rachele Stefania, Valentina Gaidano, Alessandro Cignetti, Michela Spinelli, Carmen Fava, Mahin Nikougoftar Zarif, Sara Galimberti, Ester Pungolino, Mario Annunziata, Luigia Luciano, Giorgina Specchia, Monica Bocchia, Gianni Binotto, Massimiliano Bonifaccio, Bruno Martino, Patrizia Pregno, Fabio Stagno, Sabina Russo, Silvio Aime, Paola Circosta, Giuseppe Saglio

Electronic Supplementary Information

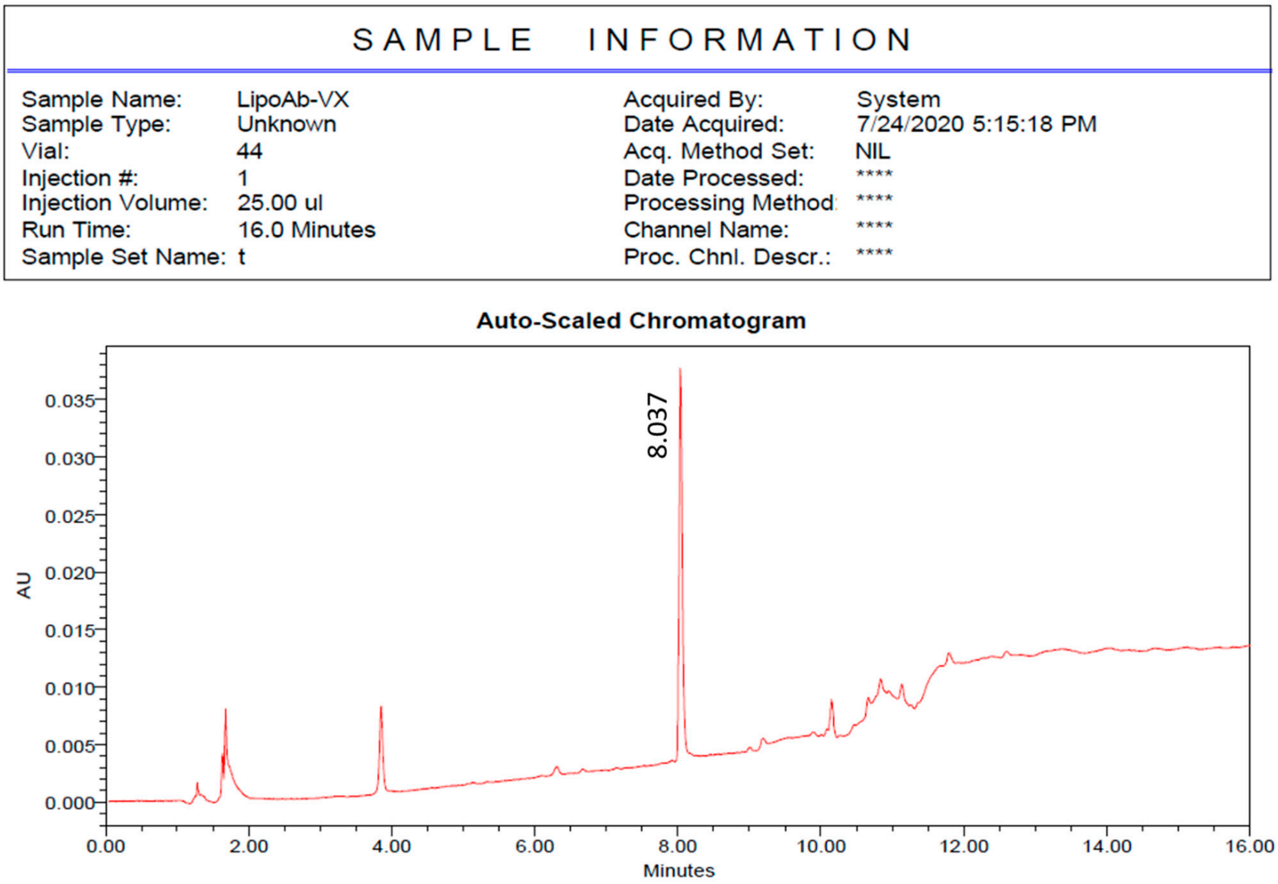

Figure S1. HPLC chromatogram of venetoclax supernatant extracted from immunoliposome at 254 nm, t_R= 8.03 min.

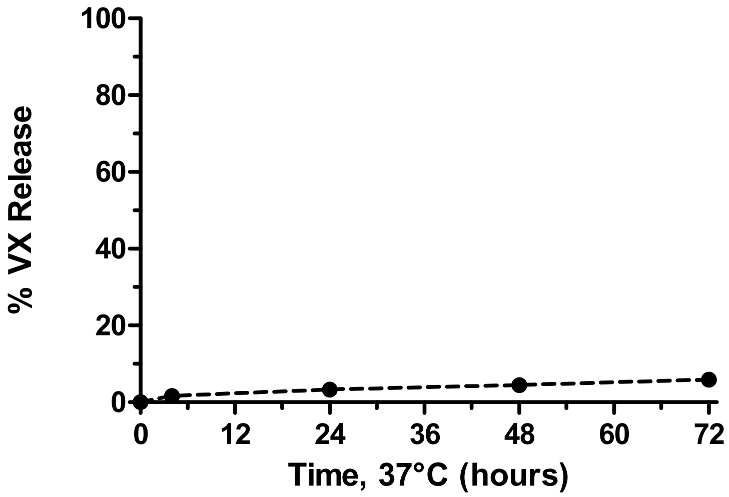

Figure S2. % Venetoclax cumulative release from IL-VX measured at 37°C, at different time points. IL-VX were placed in dialysis membrane; various aliquots of IL-VX were collected at different time points and analyzed by HPLC. Results are expressed as mean ± SD.
